# Book Reviews

**Published:** 1912-03

**Authors:** 


### BOOK REVIEWS

PRACTICAL ELECTRO-THERAPEUTICS AND X-RAY THERAPY. By J. M. Martin, M. D., Professor of Electro-Therapeutics and X-ray methods in the Medical Department of Southwestern University, Dallas, Texas. Containing 219 illustrations. Published by C. V. Mosby Co., St. Louis, Mo.

This book was written for the student and general practitioner and has been simplified so as to eliminate the deep theoretical part. It should be of great value to those interested in this important field of medicine.

---

INFECTIONS OF THE HAND.. A guide to the surgical treatment of the acute and chronic suppurative processes in the fingers, hand and forearm. By Allen B. Kanavel, M. D., Asst. Prof. of Surgery, Northwestern University Medical School, Chicago. Illustrated with 133 engravings. Published by Lea & Febiger, Philadelphia.

It is indeed unusual to find a more satisfactory and scientific work upon a surgical subject than is this book on infections of the hand. It is a well balanced admixture of experimental and anatomical investigations with every-day practical and useful facts.

---

DORLAND'S AMERICAN ILLUSTRATED MEDICAL DICTIONARY. A new and complete dictionary of terms used in Medicine, Surgery, Dentistry, Pharmacy, Chemistry, Veterinary Medicine, Nursing, Biology, and kindred branches; with new and elaborate tables. Sixth Revised Edition. Edited by W. A. Newman Dorland, M. D. Large octavo of 986 pages, with 323 illustrations, 119 in colors. Containing over 7,000 more terms than the previous edition. Philadelphia and London: W. B. Saunders Company, 1911. Flexible leather, $4.50 net; thumb indexed, $5.00 net.

Many important improvements have been made in this edition of Dorland's well-known dictionary and many new words are included which are not found in other medical dictionaries.

---

VETERINARY BACTERIOLOGY. By Robert E. Buchanan, Ph. D., Professor of Bacteriology in the Iowa State College of Agriculture and Mechanic Arts, Division of Veterinary Medicine. Octavo of 516 pages, with 214 illustrations. Philadelphia and London: W. B. Saunders Company, 1911. Cloth, $3.00.

This volume is a revision of the lectures on veterinary bacteriology given during the past six years to classes in the Division of Veterinary Medicine in the Iowa State College.

---

MICROSCOPY, BACTERIOLOGY AND HUMAN PARASITOLOGY. By P. E. Archinard, A. M., M. D., Bacteriologist, Louisiana State Board of Health and City Board of Health, New Orleans. New (2d) edition, thoroughly revised. 12mo, 267 pages, with 100 engravings and 6 plates. Cloth $1.00 net. The Medical Epitome Series. Lea & Febiger, Publishers, Philadelphia and New York, 1912.

As a concise presentation of the essential points of Bacteriology and Microscopy this little work has won the favor of students and practitioners. In its new edition the scope has been broadened to include some of the protozoa, an improvement which should increase its usefulness to the practicing physician and to the advanced student. The features which gained for it the approbation of its readers have been continued in the present very thorough revision.

